# Efgartigimod as a promising add-on therapy for myasthenic crisis: a prospective case series

**DOI:** 10.3389/fimmu.2024.1418503

**Published:** 2024-07-29

**Authors:** Jie Song, Haiyan Wang, Xiao Huan, Qilong Jiang, Zongtai Wu, Chong Yan, Jianying Xi, Chongbo Zhao, Huiyu Feng, Sushan Luo

**Affiliations:** ^1^ Huashan Rare Disease Center and Department of Neurology, Huashan Hospital, Shanghai Medical College, National Center for Neurological Disorders, Fudan University, Shanghai, China; ^2^ Department of Neurology, The First Affiliated Hospital, Sun Yat-Sen University, Guangzhou, China; ^3^ Department of Neurology, The First Affiliated Hospital of Guangzhou University of Chinese Medicine, Guangzhou, China; ^4^ Faculty of Biology, University of Cambridge, Cambridge, United Kingdom

**Keywords:** myasthenic crisis, efgartigimod, ventilation, respiratory muscle, antibody, immunoglobulin G

## Abstract

**Introduction:**

Efgartigimod is effective and well-tolerated in patients with anti-acetylcholine receptor (AChR) antibody-positive generalized myasthenia gravis (MG). However, the therapeutic potential and the safety profile of efgartigimod in myasthenic crisis (MC) remained largely unknown.

**Methods:**

This is an observational, prospective, multicenter, real-world study to follow 2 MC patients who initiated efgartigimod as a first-line rescue therapy and 8 cases who used it as an add-on therapy. Baseline demographic features and immunotherapies were collected, and the MG-activities of daily living (MG-ADL) scale was evaluated every week since efgartigimod treatment for 8 weeks. Additionally, serum IgG and anti-AChR antibody levels and the peripheral CD4^+^ T lymphocytes were measured before and after one cycle of treatment.

**Results:**

Ten patients with MC were enrolled in the study, including 9 anti-AChR antibody positive and 1 anti-muscle-specific kinase (MuSK) positive. All patients were successfully weaned from the ventilation after receiving efgartigimod treatment, with a length of 10.44 ± 4.30 days. After one cycle of infusions, the MG-ADL score reduced from 15.6 ± 4.4 at the baseline to 3.4 ± 2.2, while the corticosteroid dose was tapered from 55.0 ± 20.7 mg to 26.0 ± 14.1 mg. The proportions of regulatory T cells and naïve T cells (% in CD4^+^ T) significantly decreased post-efgartigimod treatment (5.48 ± 1.23 vs. 6.90 ± 1.80, *P*=0.0313, and 34.98 ± 6.47 vs. 43.68 ± 6.54, *P*=0.0313, respectively).

**Conclusion:**

These findings validated the rapid action of efgartigimod in facilitating the weaning process with a good safety profile in patients with MC.

## Introduction

Myasthenia gravis (MG) is a chronic autoimmune neuromuscular disorder caused by antibodies attacking the neuromuscular junction (NMJ). The involvement in the respiratory muscle of MG patients usually leads to the most severe life-threatening state, namely myasthenic crisis (MC), requiring noninvasive and/or invasive ventilation. MC is the most prevalent cause of death among MG patients, and the mortality rates range from 5% to 12% (1, 2). With the development of prompt rescue therapies utilizing intravenous immunoglobulin (IVIg) or therapeutic plasma exchange (TPE), the in-hospital outcome has been substantially improved (3). However, not all MC cases respond well to rescue therapies, and prolonged endotracheal intubation is associated with a higher risk of developing ventilator-associated complications (4). Thus, given the requirement for intensive care and hospitalization and the associated burden of disease, this highlights the importance of introducing new therapies with fewer side effects and rapid onset of action for MC.

Anti-acetylcholine receptor (AChR) antibodies, primarily in the immunoglobulin subclass (IgG1 and IgG3), play a pivotal role in the immunopathogenesis of MG. On the other hand, anti-muscle specific kinase (MuSK) antibodies are identified in approximately 5-10% of MG patients, mainly encompass IgG4 (5, 6). IgG is the predominant class of antibodies, accounting for approximately 75-80% of the total immunoglobulin pool (7). The neonatal Fc receptors (FcRn) uniquely bind both IgG and albumin, thus preventing their breakdown by reducing the lysosomal degradation and releasing outside the cell (8). Efgartigimod is a modified Fc fragment derived from human IgG1, which has been specifically engineered to enhance the binding affinity to FcRn. Multiple doses of efgartigimod efficiently cleared IgG levels while preserving albumin (9). The ADAPT trial showed a promising response in AChR+ gMG patients (9). However, individuals with Myasthenia Gravis Foundation of America (MGFA) class V were excluded from the study. As a result, the therapeutic potential of efgartigimod in treating MC remains largely unknown.

The present study prospectively followed a small case series of patients with MC who initiated efgartigimod as a rescue therapy. We attempted to evaluate the efficacy of efgartigimod in facilitating the weaning process and the safety profile for patients with MC.

## Methods

This is an observational, prospective, multicenter, real-world study. The participants were enrolled during the MC with a previously confirmed MG diagnosis. The diagnosis was made according to the established guidelines with either positive AChR antibody, or MuSK antibody, or double negative for antibody testing but positive repetitive nerve stimulation (RNS) at low frequency (2-5Hz), and responsive for neostigmine tests with the exclusion of congenital myasthenic syndrome and Lambert-Eaton syndrome (10). Generalized MG (gMG) was divided into the following subtypes: anti-AChR-positive (AChR+) early-onset MG (EOMG), anti-AChR-positive (AChR+) late-onset MG (LOMG), thymoma-associated MG (TMG), anti-MuSK-positive (MuSK-MG), and seronegative MG (SNMG) (11). Maintenance therapies with IVIg or TPE were defined as repeated IVIg and/or plasma exchange (PE), or lymphocyte plasma exchange (TPE) for the past three months. MC denotes a status of severe myasthenic weakness requiring intubation or noninvasive respiratory support. Refractory MC was defined as those who had repeated failure for rescue therapies, including monotherapy or a combination of the following: IVIg and TPE during the crisis, but still not able to wean from ventilation for longer than 30 days.

To explore the safety and efficacy of efgartigimod in facilitating the weaning process of MC, we only enrolled patients who had initiated efgartigimod after noninvasive or invasive mechanical ventilation (MV) and had completed at least one infusion cycle. Cases with respiratory failure due to severe congestive heart failure, phrenic nerve injury, or adult respiratory distress syndrome were excluded. Efgartigimod was administered intravenously at a dose of 10mg/kg as a one-hour infusion in cycles of four weekly infusions, followed by a fixed four-week intertreatment period and observation. The treatment-emergent adverse events (TEAEs) were recorded and prospectively collected.

The primary outcome was defined as the duration of ventilation after initiating efgartigimod treatment (days). Secondary outcomes included the safety profile during the crisis, the change in MG activities of daily living (MG-ADL) scores, and the tapering of corticosteroid dose after efgartigimod initiation. The baseline clinical features were collected, such as age, sex, weight, serum antibody status, onset age, thymoma concurrence, comorbidities, and other immunotherapies before and after efgartigimod treatment. To evaluate the therapeutic efficacy of efgartigimod, MG-ADL scores were prospectively collected at baseline and each week after efgartigimod treatment until eight weeks. The time from efgartigimod initiation to the successful weaning and the total in-hospital stay were also retrieved.

Serum IgG levels before efgartigimod treatment were measured using an immunoturbidimetric assay according to the manufacturer’s instructions (Siemens, Germany). Serum antibodies against AChR and MuSK were measured using enzyme-linked immunosorbent assays (ELISA, Euroimmun, Lübeck, Germany). The serum from participants at crisis was obtained and sent for AChR antibody testing. If the results were negative for anti-AChR antibody testing, the MuSK antibody would be tested next. In this prospective cohort, eight participants had repeated serum IgG measurements and six had repeated anti-AChR antibody measurements at baseline and after one cycle of efgartigimod treatment.

Flow cytometry immunophenotyping was performed to identify peripheral CD4^+^ T cell subsets in participants’ blood samples. This was conducted before efgartigimod treatment and after one cycle of treatment. We followed the protocol of sample handling described in our previous study (12). Then the frequency of CD4^+^T subsets was identified as follows: Regulatory T cells (Tregs): CD25^hi^CD127^dim^; Th1: CXCR3^+^CCR6^-^; Th2: CXCR3^-^CCR6^-^; Th17: CXCR3^-^CCR6^+^; Tfh: CXCR5^+^; naïve T cells (Tnaive): CCR7^+^CD45RA^+^; T effector memory cells (TEM): CCR7^-^CD45RA^-^, T central memory cells (TCM): CCR7^+^CD45RA^-^, and the T effector memory RA+ (TEMRA): CCR7^-^CD45RA^+^.

## Results

We enrolled ten patients with MC who initiated efgartigimod as a rescue therapy during the crisis recruited from 21^st^ September 2023 to 8^th^ February 2024 from three University hospitals. At admission, the age was 55.5 ± 17.1 years old with a female-to-male ratio of 1:1. The duration of the disease was 3.9 ± 8.1 years from the onset to the current crisis. The gMG subtypes included EOMG (n=2), TMG (n=6), LOMG (n=1) and MuSK-MG (n=1). The proportion for thymectomy was 60% (6/10). The immunotherapies before the current MC included corticosteroid (100%,10/10), tacrolimus (50%,5/10), azathioprine (10%,1/10), mycophenolate mofetil (MMF) (10%,1/10), and IVIg and/or TPE maintenance (20%, 2/10). After MC development, four patients required biphasic positive airway pressure (BIPAP), two underwent tracheal intubation, and four required MV after tracheotomy (details in **Table 1**).

**Table 1:** Baseline clinical features for patients who were treated with efgartigimod during MC, n=10.

Patient No.	Sex	Age	Antibody	gMG subtype	Disease Duration, years	Thymoma	Thymectomy	Combined IS	Duration from this crisis to efgartigimod initiation, days	Duration of ventilatory support, days
1	F	73	AChR	TMG	0.5	Y	Y	Corticosteroid	1	6
2	F	59	AChR	EOMG	26	N	N	Corticosteroid	30	39
3	F	42	AChR	TMG	0.5	Y	Y	Corticosteroid Tacrolimus	57	70
4	M	67	AChR	TMG	0.5	Y	Y	Corticosteroid Tacrolimus IVIg and/or TPE	10	12
5	M	45	AChR	TMG	4	Y	Y	Corticosteroid Tacrolimus Mycophenolate mofetil	2	14
6	M	75	AChR	TMG	0	Y	Y	Corticosteroid	0	14
7	M	41	AChR	TMG	0.5	Y	Y	Corticosteroid Tacrolimus	146	156
8	F	76	AChR	LOMG	7	N	N	Corticosteroid Azathioprine IVIg and/or TPE	26	40
9	F	26	AChR	EOMG	0	N	N	Corticosteroid	6	20
10	F	51	MUSK	MUSK	0	N	N	Corticosteroid Tacrolimus	2	12

MC, myasthenic crisis; EOMG, early-onset MG; LOMG, late-onset MG; TMG, thymoma-associated MG; MuSK-MG, muscle-specific tyrosine kinase; IS, immunosuppressants; IVIg, intravenous immunoglobulin; PE, plasma exchange.

Eight patients had at least one cycle of IVIg or TPE before the initiation of efgartigimod with an intermission of over two weeks. Two TMG patients (Patient 1 and Patient 6) with very late onset age received efgartigimod as the first-line rescue therapy for MC. One patient (Patient 7) was very refractory to the conventional immunotherapies and difficult to wean from the ventilation for over five months, and thus was defined as refractory MC.

**Figure 1:**
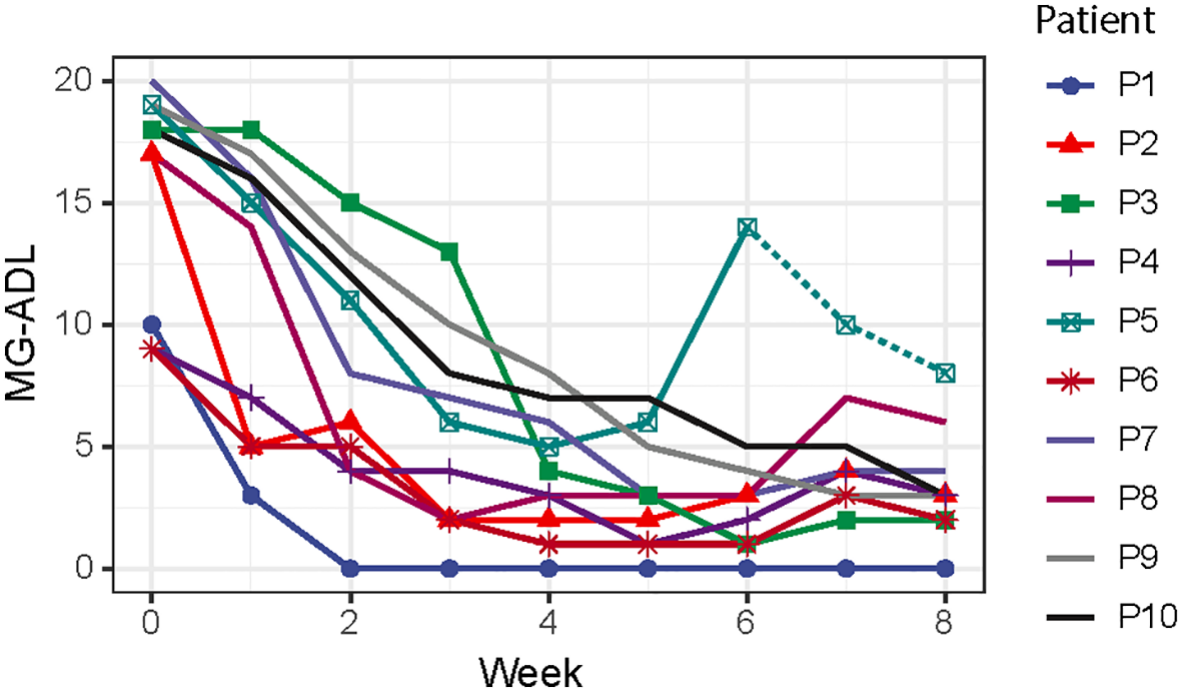
The assessments of MG-ADL scores from starting the efgartigimod treatment to eight weeks after treatment. (MG-ADL, myasthenia gravis-activities of daily living).

After efgartigimod initiation, all patients were successfully weaned from the ventilation in 10.44 ± 4.30 days, and the overall hospital stay was 33.1 ± 24.3 days. The MG-ADL score reduced from 15.6 ± 4.4 at the baseline to 3.4 ± 2.2 after one cycle of efgartigimod (**Figure 1**). Patient 5 showed an improvement after one cycle of efgartigimod, but unfortunately experienced MG exacerbation after an upper respiratory infection. The corticosteroid dose of all patients was tapered from 55.0 ± 20.7 mg at the baseline to 26.0 ± 14.1 mg after one cycle of efgartigimod treatment. Two patients who received efgartigimod as the first-line rescue therapy obtained a rapid response and successfully weaned off from BIPAP (5 days) and oral intubation (14 days), respectively, avoiding the need for tracheostomy.

Efgartigimod was well-tolerated in this cohort with MC. We initiated efgartigimod during the stable stage of ventilation-associated pneumonia in 6 cases, who were already treated with ceftazidime or piperacillin-tazobactam intravenously. Upon starting the treatment, patients had white blood cell count of 9.7 ± 10.1*10^9^/L and serum C protein level of 10.8 ± 9.8 mg/L. After one cycle of efgartigimod, the white blood cell count decreased to 6.9 ± 2.8*10^9^/L and the serum C protein level decreased to 1.2 ± 1.5 mg/L. During the observation period, four adverse events were reported among the ten participants, including upper respiratory infection (n=1), headache (n=1) and urinary tract infection (n=2).

**Figure 2:**
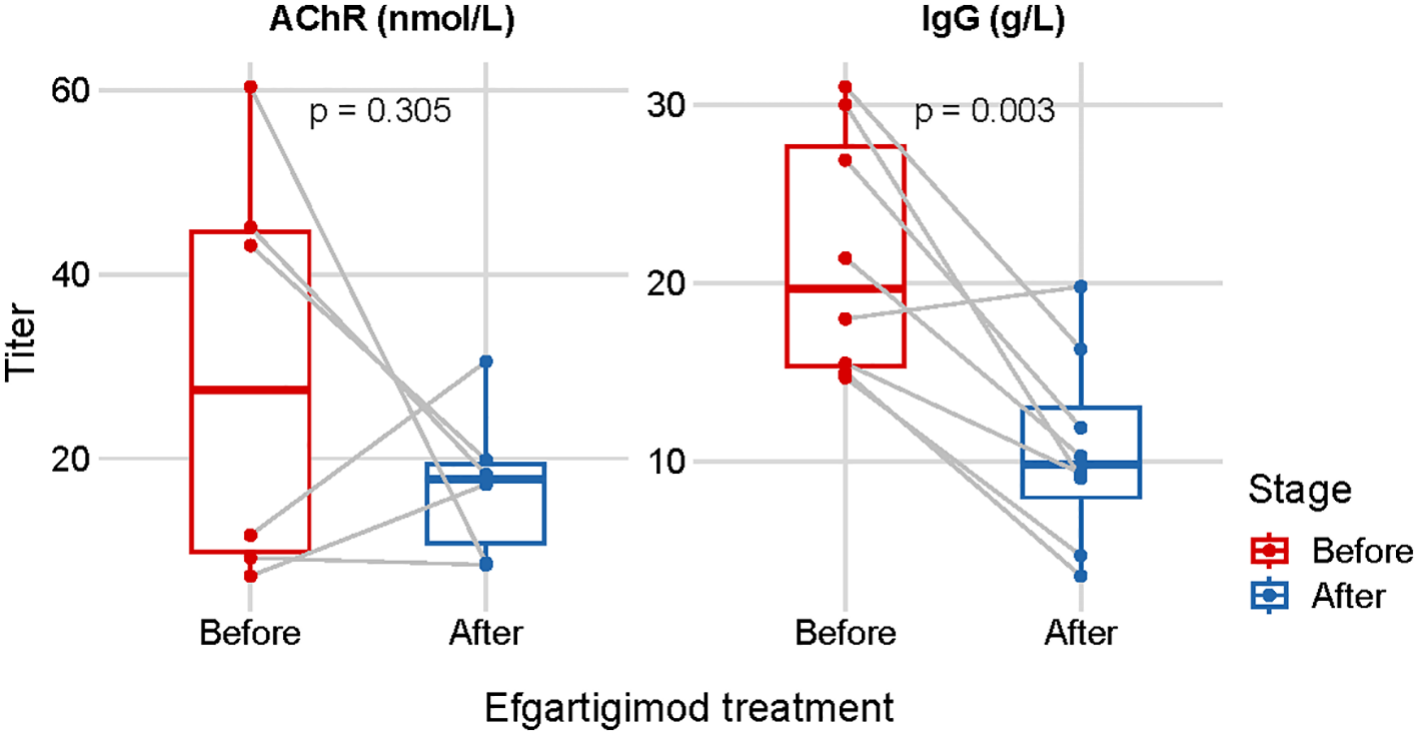
Repeated measurements of the serum IgG (n=8) and anti-AChR antibody levels (n=6) before- and after one cycle of efgartigimod treatment.

The administration of efgartigimod reduced both total IgG and specific autoantibodies. We measured serum IgG (n=8) and anti-AChR antibody levels (n=6) before and after efgartigimod treatment, along with weaning success longitudinally. The serum IgG levels were reduced by 50.9% from 21.6 ± 6.8 g/L (before treatment) to 10.6 ± 5.4 g/L (*p* = 0.003). Anti-AChR antibody levels were reduced by 42.0% from baseline values of 29.5 ± 22.9 nmol/L to 17.1 ± 8.2 nmol/L (*p* = 0.305). (**Figure 2**).

In addition to the serum IgG and antibody levels, the peripheral CD4^+^ T profile was measured using a previously characterized flow cytometry panel (n=6). The proportions of Tregs (% in CD4^+^ T) and Tnaive cells significantly decreased after efgartigimod treatment (5.48 ± 1.23 vs. 6.90 ± 1.80%, *P*=0.0313, and 34.98 ± 6.47 vs. 43.68 ± 6.54%, *P*=0.0313, respectively). However, other CD4^+^ T subsets remained unchanged, including Th1, Th2, Th17, Tfh, TEM, TCM, and TEMRA cells (**Table 2**).

**Table 2:** Changes in peripheral CD4^+^T profile of MC patients before and after efgartigimod treatment.

CD4^+^ T	MC before efgartigimod treatment (n=6)	After efgartigimod treatment (n=6)	*P* value
Th1, % in CD4^+^T	14.56 ± 7.47 (9.27-28.4)	11.09 ± 7.34 (1.19-20.5)	0.8182
Th2, % in CD4^+^T	53.68 ± 3.01 (48.6-57.3)	53.77 ± 8.57 (44.5-66.2)	>0.99
Th17, % in CD4^+^T	22.90 ± 8.36 (8.87-30.6)	27.84 ± 11.42 (9.83-42.3)	0.3125
Tfh, % in CD4^+^T	19.75 ± 6.60 (11.2-26.2)	19.21 ± 9.52 (6.4-28.1)	0.8438
Treg, %in CD4^+^T	6.90 ± 1.80 (4.13-9.78)	5.48 ± 1.23 (3.18-6.45)	0.0313*
Tnaive, % in CD4^+^T	43.68 ± 6.54 (35.7-50.3)	34.98 ± 6.47 (24.9-44.2)	0.0313*
TEM, % in CD4^+^T	10.81 ± 4.26 (6.31-17)	13.24 ± 5.00 (6.75-20.2)	0.2188
TCM, % in CD4^+^T	38.47 ± 6.66 (25.5-43.8)	41.60 ± 11.56 (20.8-53.7)	0.4375
TEMRA, % in CD4^+^T	7.24 ± 7.62 (0.77-21.8)	10.26 ± 13.20 (1.41-36.7)	0.4375

*P<0.05.

MC, myasthenic crisis; Th, T helper; TCM, T central memory; TEM, T effector memory; TEMRA, T effector memory RA+; Tnaive, naïve T; Tfh, T follicular helper; Treg, regular T. The bold values represent the p-values for the statistical analysis of Treg cells and naive T cells before and after treatment.

## Discussion

The primary goal of treating patients with MC is to efficiently improve respiratory muscle strength, wean them off ventilators, and reduce the duration of overall ICU stay. Unfortunately, an urgent requirement existed for a new rescue therapy to address the needs of patients with MC who do not respond to, or are intolerant to, IVIg or TPE, which are currently efficacious treatments for MG acute exacerbations and MC (13). Efgartigimod is a recently approved FcRn antagonist for treating gMG, yet it has not been extensively applied in patients during the MC. This study reported the safety and efficacy profile of efgartigimod in ten patients from the crisis to the post-crisis stages.

Although MC is closely associated with pneumonia, mostly ventilation-associated pneumonia (VAP), the safety profile of FcRn antagonists in this cohort has been generally favorable. This may be potentially attributed to the early intervention of antibiotics, which may be a confounder. However, there is still a concern regarding the increased risk of infections caused by the therapeutic inhibition of FcRn to remove the pathogenic and nonpathogenic IgG across the epithelial cells lining the respiratory tract (14, 15). Furthermore, from animal studies, FcRn participates in immune responses to several bacterial and viral infections (16). Therefore, collaborative efforts are needed to provide adequate ventilatory support, anti-microbial treatments, and intensive care to prevent the development of pneumonia and hypogammaglobulinemia while treating MC patients.

The therapeutic efficacy of efgartigimod in MC has been demonstrated, as evidenced by successful weaning within an average duration of less than two weeks. We hypothesized the possible underlying mechanisms as 1) A significant reduction in anti-AChR antibody levels using FcRn blocking, which is associated with an improved clinical status (17); and 2) Inhibition of immune cell activation. Within the immune compartment, FcRn expression is particularly high in myeloid cells such as monocytes, tissue-resident macrophages, dendritic cells (DCs) and neutrophils; in lymphocytes, low levels of FcRn are present in B cells and no FcRn expression has been detected in T cells or natural killer (NK) cells (18). These findings are consistent with our previous identification of innate immune activation, which is probably the cause of peripheral hypercytokinemia during MC (12, 19).

Noticeably, Patient 7 (TMG) and Patient 8 (LOMG) had inversely elevated serum levels of anti-AChR antibodies, while the IgG levels decreased, or remained stable after efgartigimod treatment. The following reasons may explain this unexpected increase in anti-AChR antibody levels. First, the corticosteroid dose of all patients was tapered from 55.0 ± 20.7 mg at the baseline to 26.0 ± 14.1 mg after one cycle of efgartigimod treatment. These would probably catalyze an immune response, although the clinical MG-ADL score remarkably decreased. Secondly, the pathogenicity of anti-AChR antibodies associated with NMJ impairment cannot be simply evaluated by peripheral antibody levels. A recent study supported that receptor clustering and pathogenic complement activation in MG depend on synergy between antibodies with multiple subunit specificities (20). Even individual autoantibody clones can mediate multi-pathogenicity (21). In contrast, the changes in serum IgG levels were more consistent with the clinical outcome as measured by MG-relevant scores and days of ventilatory support in this MC cohort. Future studies are required to advance the measurement of multifaceted “pathogenic” mechanisms”.

Beyond the decreased levels in AChR antibody and IgG after treatment, we also observed a previously undescribed decrease in peripheral naïve CD4^+^ T cell subsets among patients after efgartigimod treatment. Apart from the binding with IgG and albumin and protecting them from degradation in lysosomes, FcRn plays a role in the presentation of antigen presentation, by dendritic cells (DCs) to CD4^+^ T cells, which are complexed with IgGs (22). Impaired antigen presentation from DCs, for instance, by FcRn blocking, may impede the naïve T cell activation and expansion, as well as the differentiation from CD4^+^ T cells into effector T cells (23). In this regard, future longitudinal cohort studies on MG are expected to better explore the impact of efgartigimod on the changes of peripheral immune cells.

Conventional rescue therapies for treating MC include IVIg and TPE, but both have drawbacks. IVIg administration can cause side-effects such as aseptic meningitis, headaches, increased propensity to thrombosis and renal impairment and cardiac failure (24). TPE is a therapeutic approach employed in MG treatment since 1976 (25). The effects of TPE last for 2-4 weeks, which requires specialized equipment, central venous access, and supervision. In patients who deteriorate following TPE, the presence of antibody overshoot may exist (26). However, tissue IgG is redistributed between the TPE sessions, and the serum IgG rises again. To address these issues, there is a need for agents that mimic the role of TPE or IVIg but have a sustained effect and fewer side effects.

As the first approved FcRn antagonist for treating MG, efgartigimod has a higher affinity to block FcRn in comparison to the competitive saturation from IVIg, which has been mainly shown in *in vitro* studies using transfected endothelial cells expressing human FcRn-GFP (27). Unlike TPE, which depletes all immunoglobulin subtypes, FcRn selectively binds IgG, including pathogenic autoantibodies, without binding or depleting other immunoglobulins. This selectivity and the IgG half-life (approximately 21 days) means that continuous FcRn blockade with FcRn inhibitors is possible once a steady state has been achieved, leading to more sustained IgG depletion (27). This contrasts with TPE, which achieves rapid but transient and nonselective immunoglobulin depletion, and requires multiple sequential procedures to achieve a similar level of IgG reduction. Furthermore, FcRn is widely expressed in various tissues and cells including epithelia, endothelia, hemopoietic cells, intestinal cells, kidney, liver, and placenta (28). FcRn blockade inhibits the IgG transfer among various tissues and cells more directly among various tissues and cells than the primary removal of the circulating immunoglobulins.

This study has some limitations that can be optimized for further research. First, efgartigimod was mostly applied as the second-line rescue therapy in this case series. The safety and efficacy of efgartigimod in treating MC should be explored as the first-line rescue therapy in a well-designed prospective cohort with larger sample size and head-to-head comparisons to the TPE or IVIg. Additionally, we did not include the patients who failed to complete the entire cycle of efgartigimod from the crisis. This may have included some patients who were refractory and switched to other rescue therapies. Third, the overlapping efficacy of the rescue therapies and the combined immunosuppressants (IS) may lead to an overamplification of the therapeutic efficacy. The heterogeneity of the participants, including those with thymoma-associated exacerbations further complicates the analysis of the changes in serum IgG and anti-AChR antibody levels. Last, the confirmation of serological diagnosis should be made based on radioimmunoassays or cell-based assays.

## Conclusion

In conclusion, our preliminary study supports the role of efgartigimod as a promising rescue therapy, especially for patients with refractory MC or who cannot tolerate conventional rescue therapies. The rapid action of efgartigimod in facilitating the weaning process and its good safety profiles allow future cohort studies to explore its applications in patients with acute exacerbations and MC.

## Data availability statement

The raw data supporting the conclusions of this article will be made available by the authors, without undue reservation.

## Ethics statement

The studies involving humans were approved by Huashan Hospital, Fudan University. The studies were conducted in accordance with the local legislation and institutional requirements. The participants provided their written informed consent to participate in this study.

## Author contributions

JS: Writing – original draft, Methodology, Data curation, Conceptualization. HW: Writing – original draft, Investigation, Conceptualization. XH: Writing – review & editing, Methodology, Investigation, Data curation. QJ: Writing – review & editing, Methodology, Investigation, Data curation. ZW: Writing – original draft, Software, Formal analysis. CY: Writing – review & editing, Methodology, Investigation, Data curation. JX: Writing – review & editing, Supervision, Data curation. CZ: Writing – review & editing, Funding acquisition. HF: Writing – review & editing, Supervision, Investigation, Conceptualization. SL: Writing – review & editing, Visualization, Validation, Data curation, Conceptualization.
